# Restriction of dietary protein decreases mTORC1 in tumors and somatic tissues of a tumor-bearing mouse xenograft model

**DOI:** 10.18632/oncotarget.5180

**Published:** 2015-09-03

**Authors:** Dudley W. Lamming, Nicole E. Cummings, Antonella L. Rastelli, Feng Gao, Edda Cava, Beatrice Bertozzi, Francesco Spelta, Roberto Pili, Luigi Fontana

**Affiliations:** ^1^ Department of Medicine, University of Wisconsin-Madison, Madison, WI, USA; ^2^ William S. Middleton Memorial Veterans Hospital, Madison, WI, USA; ^3^ Endocrinology and Reproductive Physiology Graduate Training Program, University of Wisconsin-Madison, Madison, WI, USA; ^4^ Division of Oncology, Washington University in St. Louis, MO, USA; ^5^ Division of Biostatistics, Washington University in St. Louis, MO, USA; ^6^ Division of Geriatrics and Nutritional Sciences, Washington University in St. Louis, MO, USA; ^7^ Department of Experimental Medicine, University of Rome “La Sapienza”, Italy; ^8^ Department of Medicine, University of Verona, Italy; ^9^ Division of Hematology/Oncology, Department of Medicine, Indiana University School of Medicine, Indianapolis, IN, USA; ^10^ Department of Clinical and Experimental Sciences, Brescia University Medical School, Brescia, Italy; ^11^ CEINGE Biotecnologie Avanzate, Napoli, Italy

**Keywords:** protein restriction, mTOR, mice, cancer, aging

## Abstract

Reduced dietary protein intake and intermittent fasting (IF) are both linked to healthy longevity in rodents, and are effective in inhibiting cancer growth. The molecular mechanisms underlying the beneficial effects of chronic protein restriction (PR) and IF are unclear, but may be mediated in part by a down-regulation of the IGF/mTOR pathway. In this study we compared the effects of PR and IF on tumor growth in a xenograft mouse model of breast cancer. We also investigated the effects of PR and IF on the mechanistic Target Of Rapamycin (mTOR) pathway, inhibition of which extends lifespan in model organisms including mice. The mTOR protein kinase is found in two distinct complexes, of which mTOR complex 1 (mTORC1) is responsive to acute treatment with amino acids in cell culture and *in vivo*. We found that both PR and IF inhibit tumor growth and mTORC1 phosphorylation in tumor xenografts. In somatic tissues, we found that PR, but not IF, selectively inhibits the activity of the amino acid sensitive mTORC1, while the activity of the second mTOR complex, mTORC2, was relatively unaffected by PR. In contrast, IF resulted in increased S6 phosphorylation in multiple metabolic tissues. Our work represents the first finding that PR may reduce mTORC1 activity in tumors and multiple somatic tissues, and suggest that PR may represent a highly translatable option for the treatment not only of cancer, but also other age-related diseases.

## INTRODUCTION

Chronic restriction of dietary protein intake and intermittent fasting are both strongly associated with health and longevity in rodents [1–4]. Epidemiological data suggest high protein intake is associated with a 75% increase in overall mortality in humans, as well as a 4-fold increase in cancer death [1, 5]. Protein restriction (PR) and intermittent fasting (IF) regimens also show beneficial effects on rodent models of age-related diseases, including cancer and Alzheimer's disease [6–8]. However, the molecular mechanisms underlying the beneficial effect of PR and IF diets on metabolic health and longevity have not been clearly established, although PR and IF diets significantly reduce circulating levels of IGF-1 (Insulin-like growth factor 1), a potent activator of the mTOR (mechanistic Target Of Rapamycin) signaling pathway [9, 10].

Inhibition of the insulin/IGF-1/PI3K/mTOR signaling pathway either genetically or by treatment with the FDA-approved pharmaceutical rapamycin promotes longevity and inhibits tumor growth in model organisms [7, 11–14]. Unfortunately, the potentially serious side effects of rapamycin and rapamycin analogs in humans may preclude the use of these compounds to prevent or delay age-related diseases [15, 16]. The mTOR protein kinase is found in two complexes, mTOR complex 1 (mTORC1) and mTORC2, each with distinct substrates; genetic studies have demonstrated that inhibition of mTORC1 signaling is sufficient to extend lifespan, while inhibition of mTORC2 reduces insulin sensitivity and decreases male lifespan [17]. We and others have demonstrated that chronic treatment of mice with rapamycin results in the disruption of both mTOR complexes [18, 19]. Disruption of mTORC2 contributes to the negative metabolic and immunological side-effects of rapamycin, and we have proposed that specifically inhibiting mTORC1 would promote longevity and prevent age-associated diseases (e.g. cancer) with many fewer side effects than rapamycin, which inhibits both complexes [16].

In this study we compared the effects of chronic or intermittent reduction of protein intake on tumor growth in a xenograft mouse model of breast cancer. As amino acid levels in serum and tissue are regulated by dietary protein intake [20], and mTORC1 is acutely sensitive to amino acids, which activate mTORC1 by promoting its localization to the lysosome and its interaction with Rheb, we also examined mTORC1 and mTORC2 signaling not only in the xenograft tumors but also in several key metabolic tissues (liver, skeletal muscle, heart muscle and adipose tissue) of mice fed either a 21% and 7% protein diet *ad libitum*, or maintained on an IF regimen utilizing either a 21% or 7% protein diet.

## RESULTS

### Protein restriction and intermittent fasting inhibit tumor growth in a human breast cancer model

**Figure 1:**
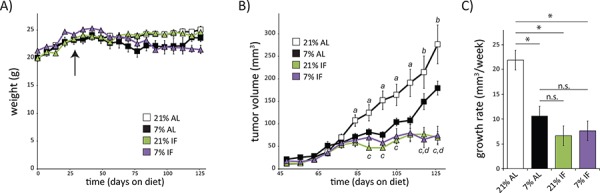
A low protein diet or intermittent fasting regimen slows tumor growth rate in a mouse model of breast cancer **A.** Weights of 6 week old female mice placed on diets containing either 21% or 7% protein, which were fed either *ad libitum* (AL) or with intermittent, alternate day fasting (IF). Arrow indicates time of tumor implantation on day 29. **B.** Tumor volumes were measured weekly starting 20 days after tumor cell implantation. (*n* = 7–10 mice per group, *a* = *p* ≤ 0.05, *b* = *p* ≤ 0.09, 21% AL vs. all other groups; *c* = *p* < 0.008, 7% AL vs. 21% IF; *d* = *p* < 0.006, 7% AL vs 7% IF). Data in A-B for AL fed mice was previously published in part [7] and is shown here for comparison. **C.** Growth rates of tumors in mice on each indicated diet was calculated using the method of Laird and Ware [34] from tumor volumes measured 3–12 weeks after tumor implantation (*n* = 9–10 mice per group, *p* < 0.0001).

To test the hypothesis that chronic dietary PR was more effective than intermittent fasting (IF) in inhibiting tumor growth in an animal model of human breast cancer, we acclimatized 4–6 week old female NOD-SCID mice to either a 21% or 7% (PR) protein diet, fed *ad libitum* or intermittently (IF) for 4 weeks, prior to subcutaneous implantation of WHIM16 ER+/PR-/HER2- tumor cells. Mice fed a PR diet either *ad libitum* or intermittently were approximately 10–15% lighter than mice fed a control diet by the latter half of the study, while mice fed a 21% diet intermittently were the same weight as *ad libitum* fed mice (Fig. 1A). As we have previously shown [7], the growth of WHIM16 ER+/PR-/HER2- xenografts is significantly reduced in mice fed a 7% protein diet *ad libitum* compared to a 21% protein control diet (Fig. 1B). Interestingly, we find that IF has similar effects, independent of dietary protein intake, in reducing tumor growth. The tumor growth rate in mice fed either diet intermittently (every other day) was significantly smaller than that in mice on an *ad libitum* 21% protein diet (Fig. 1C). The tumor volume of IF mice was significantly lower than of mice fed either a 21% or a 7% protein diet *ad libitum* during the second part of the study (Fig. 1B).

### Protein restriction attenuates mTOR activity in human breast cancer xenografts

The mTOR signaling pathway is a critical regulator of growth and cellular proliferation, and mutations that activate mTORC1 are linked to cancer [21–23]. mTORC1 activity is highly responsive to amino acids, which are significantly reduced in mice on a PR diet [24, 25], and mTORC1 is significantly reduced on a protein-free diet [20]. We were therefore interested in determining whether or not a 2/3rds reduction in protein intake down-regulates the mTORC1 signaling pathway in a mouse model of human breast cancer, and whether this effect was stronger than intermittent fasting, in which proteins are reduced intermittently. To test this hypothesis, we assessed the phosphorylation of S6 S240/S244, a substrate of S6K1 and a readout for mTORC1 activity. We also assessed the phosphorylation of AKT S473, a substrate of the amino acid insensitive mTORC2.

**Figure 2:**
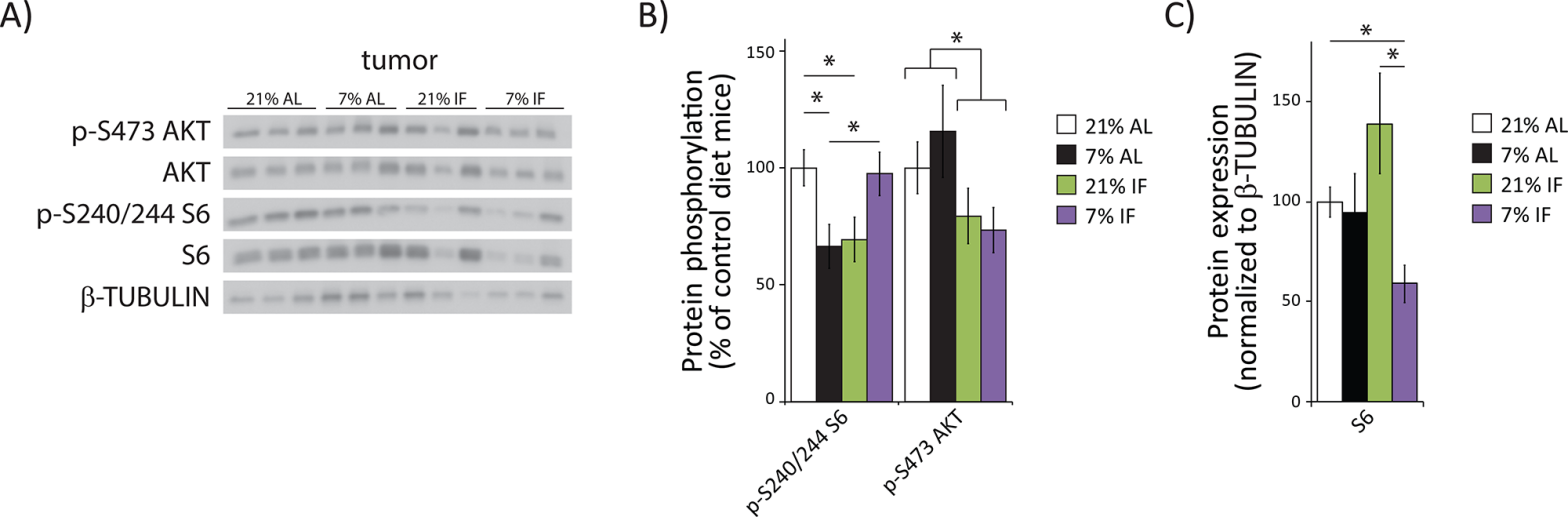
A protein restricted diet inhibits mTORC1 signaling in tumors **A.** Tissue lysates from tumor xenografts were examined for phosphorylation of S6 S240/S244 and AKT S473 by western blotting. **B.** Quantification of S6 and AKT phosphorylation, normalized to total S6 or AKT protein, was performed using NIH ImageJ. **C.** Quantification of S6, normalized to β-TUBULIN, was performed using NIH Image J (*n* = 7–10 tumors per group, * = *p* < 0.05 *t*-test following ANOVA).

As shown in Fig. 2, tumors from mice fed a PR diet *ad libitum* or mice subject to intermittent fasting of a 21% protein diet had an over 30% decrease in phosphorylated S6, indicating decreased mTORC1 activity (Fig. 2A, 2B). Interestingly, while mice intermittently fasted with a PR diet had normal levels of S6 phosphorylation (Fig. 2B), the expression of S6 itself was significantly reduced (Fig. 2C). Biogenesis of ribosomal proteins like S6 is itself mediated by mTORC1 [26, 27], suggesting that mTORC1 activity was decreased in these tumors as well. We determined that the abundance of phosphorylated S6 in the tumors of mice intermittently fasted with a PR diet relative to control, *ad libitum* mice was decreased by 44% (*p* = 0.002). In agreement with our hypothesis that reduced protein intake would specifically reduce mTORC1 activity, phosphorylation of AKT S473 was not affected by PR; however, intermittent fasting significantly decreased AKT phosphorylation (Fig. 2B).

### A low protein diet inhibits mTORC1 but not mTORC2 signaling in somatic tissues

**Figure 3:**
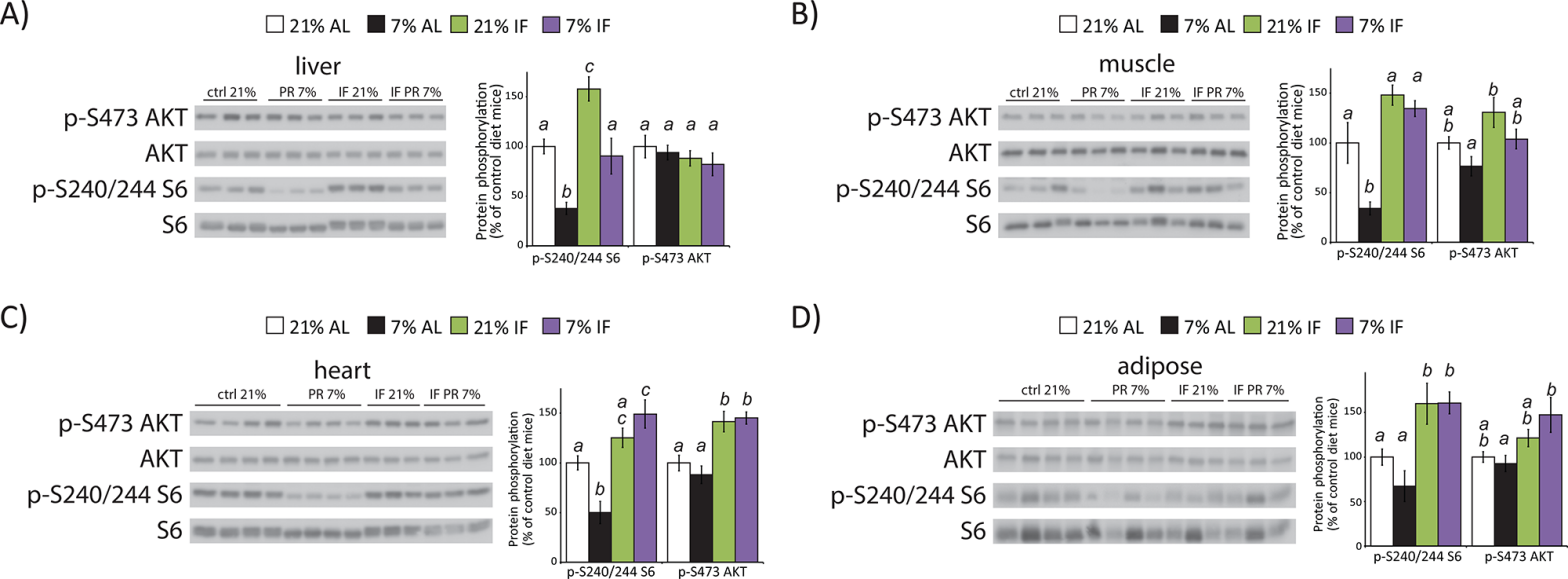
A protein restricted diet inhibits mTORC1 signaling *in vivo* Tissue lysates from **A.** liver, **B.** muscle, **C.** heart and **D.** adipose tissue were examined for phosphorylation of S6 S240/S244 and AKT S473 by western blotting. Quantification of S6 and AKT phosphorylation, normalized to total S6 or AKT protein, was performed using NIH ImageJ (*n* = 6–7 samples per group, means with the same letter are not significantly different from each other (Tukey–Kramer test following ANOVA, *p* < 0.05)).

We collected liver, skeletal muscle, heart, and adipose tissues at the end of the tumor xenograft study, and examined tissue lysates by Western blotting to test the systemic effects of protein restriction and intermittent fasting on other key metabolic tissues. We observed a dramatic effect of a PR diet on mTORC1 signaling in somatic tissues, including a greater than 60% decrease in S6 phosphorylation in liver (*p* = 0.007) and skeletal muscle (*p* = 0.0067), and a 50% decrease in heart tissue (*p* = 0.0172) (Fig. 3A–3C). We also observed a 30% decrease in S6 phosphorylation in adipose tissue, but it was not significant after correction for multiple comparisons (*p* = 0.5) (Fig. 3D). In contrast, tissues from mice subject to intermittent fasting on the 21% diet showed a significant increase of S6 phosphorylation in liver and adipose tissue, as well as a 48% increase (*p* = 0.058) in skeletal muscle (Fig. 3). Mice intermittently fasted on the 7% diet displayed increased S6 phosphorylation in heart and adipose tissue (Fig. 3). In contrast to mTORC1 activity, there was no effect of a PR diet on mTORC2 signaling as assessed by AKT S473 phosphorylation in any tissue (Fig. 3A–3D). However, intermittent fasting increased AKT S473 phosphorylation in the heart, regardless of diet composition, with a similar increase in AKT phosphorylation in 21% IF muscle and 7% IF adipose tissue.

## DISCUSSION

Diets with chronic restriction of protein intake and intermittent fasting have been demonstrated to extend lifespan and inhibit age-related diseases such as cancer. Here, we compare the effect of dietary composition and intermittent fasting on cancer growth and mTOR pathway signaling. We have found that both a reduction in dietary protein and intermittent fasting slow tumor growth rate, but have distinguishable effects on mTOR signaling both in the tumor and the somatic tissues of a tumor-bearing mouse. Within the tumor, we determined that *ad libitum* feeding of a 7% protein diet or intermittent fasting of a 21% protein diet significantly inhibits mTORC1 signaling. Previous studies have suggested that rapamycin-resistant functions of mTORC1 contribute to cell growth and cancer progression [28], and thus it will be interesting to determine if reduced dietary protein intake inhibits both rapamycin dependent and independent functions of mTORC1.

Unlike reduced dietary protein intake, we found that intermittent feeding inhibits mTORC2 as well as mTORC1. Inhibition of mTORC2 inhibits cancer progression in at least some cancers [29, 30], and thus intermittent fasting may be a particularly potent anti-cancer therapy. It is potentially important that we sacrificed the intermittently fasted mice following a day on which they enjoyed access to food; mTOR signaling in intermittently fasted mice following a fasting day might look quite different (e.g., mTORC1 signaling might be significantly decreased relative to *ad libitum* fed mice). It remains to be determined if additional, mTOR-independent mechanisms may also contribute to the anti-cancer effects of PR and IF dietary regimens.

Within the somatic tissue of the mouse, we have demonstrated that a simple dietary intervention, reduction of dietary protein intake by 2/3rds, is sufficient to significantly inhibit mTORC1 signaling in the liver, muscle, and heart tissues of NOD.Cg-*Prkdc^scid^ Il2rg^tm1Wjl^*/SzJ mice. Importantly, this occurs in the absence of mTORC2 inhibition, which is responsible for many of the negative side effects of rapamycin on metabolism and immunity [16, 17, 31–33]. Protein restriction may therefore be a highly translatable intervention for age-related diseases in humans, and the 2/3rds reduction in dietary protein we have utilized here is likely to be far more sustainable than the protein-free diets used in some recent studies. Interestingly, while intermittent fasting extends mouse lifespan, here we observed increased mTORC1 signaling in many tissues of intermittently fasted mice, suggesting that the lifespan extension observed following IF may be mTOR-independent. Future studies are warranted to determine if reduced mTORC1 signaling is the mechanism by which PR promotes health and longevity, to determine how PR impacts the circulating and tissue levels of amino acids and their metabolites, and to identify how factors such as protein quality and genetic background may influence the effect of a PR diet on mTOR signaling, longevity, and age-related diseases including cancer.

## MATERIALS AND METHODS

Antibodies to phospho-Akt S473 (4060), Akt (4691), phospho-S6 S240/S244 (2215) and S6 ribosomal protein (2217) were from Cell Signaling Technology. Protease and phosphatase inhibitor cocktail tablets were from Fisher. Other chemicals were purchased from Sigma unless noted. 2.0 mL Tough Tubes with Caps (13119–500) and 1.4 mM ceramic beads (13113–325), were purchased from Mo-Bio Laboratories, Carlsbad, CA.

### Animals

Six week old, female NOD.Cg-*Prkdc^scid^ Il2rg^tm1Wjl^*/SzJ mice were purchased from The Jackson Laboratory. Mice were housed and maintained in a sterile and pathogen free facility, in accordance with the Institutional guidelines of the Institutional Animal Care and Use Committee of the Washington University in St. Louis. WHIM16 tumor cell line was generated from a patient with ER positive/PR negative/HER2 negative breast cancer (Washington University). This tumor cell line carries also a PI3K mutation with activation of the AKT/mTOR pathway (manuscript submitted). Five million WHIM16 cells were implanted subcutaneously under the skin. All mice were operated under sedation with oxygen, isoflurane and buprenorphine. Mice were randomly grouped and placed on *ad libitum* or alternate day fasting 21% or 7% protein diets prior to tumor implantation (*n* = 10 per group).

### Feeding protocol

The two experimental diets were prepared and sterilized by irradiation by Harlan Laboratories (Madison, WI). A summary of the composition and ingredients of each diet are shown in Table 1. Animals were allowed free access to autoclaved water supply via auto-watering system. Female mice that were randomized into 21% protein diet and 7% protein diet were further randomized within each group to either receive food *ad libitum* or every other day.

**Table 1:** Calculated composition and ingredients of experimental diets used

	21% mix protein TD.10193	7% mix protein TD.10192
**Diet composition**		
Total energy value (kcal/g)	3.6	3.6
Carbohydrate (%Kcal)	58.9	73.0
Fat (%kcal)	20.1	20.2
Protein (%Kcal)	20.9	6.8
Leucine (g/kg)	25.4	8.8
Isoleucine (g/kg)	7.8	2.7
Lysine (g/kg)	16.3	4.0
Methionine (g/kg)	6.7	1.9
Cysteine (g/kg)	7.2	3
Arginine (g/kg)	6.3	2.9
Phenylalanine (g/kg)	6.6	2.4
Tyrosine (g/kg)	6.9	2.4
Histidine (g/kg)	3.4	1.4
Threonine (g/kg)	9.7	3.3
Tryptophan (g/kg)	3.4	1.0
Valine (g/kg)	8.4	3.2
**Formula (g/kg)**		
Corn	430	430
Lactalbumin (Fonterra, NZ)	177	35
DL-Methionine	2.0	0.4
Corn starch	149	287.4
Maltodextrin	100	100
Corn oil	29	32
Olive oil	29	32
Cellulose	30	30
*Mineral Mix, AIN-93G-MX	35	35
Calcium phosphate	8	8
**Vitamin Mix, Tekland	10	10

* Mineral Mix, AIN-93G-MX (No. 94046)

** Vitamin Mix, Teklad (40060)

### Tumor assessment

For tumors implanted subcutaneously, tumor sizes and body weights were recorded once a week. Tumor sizes were assessed by caliper measurements of two diameters of the tumor (longest length × shortest length = mm^2^). Tumor growth rate was calculated by summarizing the means and standard deviations for each group from 3–12 weeks following tumor cell implantation, and Laird and Ware's (1982) [34] growth curve method was used to compare the differences between groups. All the tests were two-sided and a *p*-value of 0.05 or less was taken to indicate statistical significance. The statistical analysis was performed using SAS 9.3 (SAS Institutes, Cary, NC).

### Tissue collection and immunoblotting

At the end of the xenograft experiment, at the conclusion of a full day during which both the *ad libitum* and intermittently fed groups had access to food, the food was removed at 7:30 am and the mice were then euthanized. The xenograft tumors were excised and liver, heart and skeletal muscle, and adipose tissues were collected, frozen immediately in liquid nitrogen and stored at −80°C. Tissue samples were lysed in cold RIPA buffer supplemented with phosphatase inhibitor and protease inhibitor cocktail tablets. Tissues were lysed in RIPA buffer as previously described [18] using a FastPrep 24 (M.P. Biomedicals) with bead-beating tubes and ceramic beads (Mo-Bio Laboratories), and then centrifuged for 10 minutes at 15,000 rpm. Protein concentration was determined by Bradford (Bio-Rad). 20 μg protein was separated by sodium dodecylsulpahte-polyacrylamide gel electrophoresis (SDS-PAGE) on 10% resolving gels (Life Technologies/ThermoFisher). Imaging was performed using a GE ImageQuant LAS 4000 imaging station. Quantification was performed by densitometry using ImageJ software. Statistical analysis was performed by one-way ANOVA followed by post-hoc tests using Prism (GraphPad Software).
